# Direct and Indirect Protein Interactions Link FUS Aggregation to Histone Post-Translational Modification Dysregulation and Growth Suppression in an ALS/FTD Yeast Model

**DOI:** 10.3390/jof11010058

**Published:** 2025-01-14

**Authors:** Seth A. Bennett, Samantha N. Cobos, Raven M. A. Fisher, Elizaveta Son, Rania Frederic, Rianna Segal, Huda Yousuf, Kaitlyn Chan, David K. Dansu, Mariana P. Torrente

**Affiliations:** 1 Department of Chemistry and Biochemistry, Brooklyn College, Brooklyn, NY 11210, USA; 2 Ph.D. Program in Biochemistry, The Graduate Center of the City University of New York, New York, NY 10016, USA; 3 Ph.D. Program in Chemistry, The Graduate Center of the City University of New York, New York, NY 10016, USA; 4 Neuroscience Initiative, Advanced Science Research Center, CUNY, New York, NY 10031, USA; 5 Ph.D. Program in Biology, The Graduate Center of the City University of New York, New York, NY 10016, USA

**Keywords:** FUS, amyotrophic lateral sclerosis, frontotemporal dementia, epigenetics, histone post-translational modifications, Ipl1, Rtt109, Nop1

## Abstract

Amyotrophic lateral sclerosis (ALS) and frontotemporal dementia (FTD) are incurable neurodegenerative disorders sharing pathological and genetic features, including mutations in the *FUS* gene. FUS is an RNA-binding protein that mislocalizes to the cytoplasm and aggregates in ALS/FTD. In a yeast model, FUS proteinopathy is connected to changes in the epigenome, including reductions in the levels of H3S10ph, H3K14ac, and H3K56ac. Exploiting the same model, we reveal novel connections between FUS aggregation and epigenetic dysregulation. We show that the histone-modifying enzymes Ipl1 and Rtt109—responsible for installing H3S10ph and H3K56ac—are excluded from the nucleus in the context of FUS proteinopathy. Furthermore, we found that Ipl1 colocalizes with FUS, but does not bind it directly. We identified Nop1 and Rrp5, a histone methyltransferase and rRNA biogenesis protein, respectively, as FUS binding partners involved in the growth suppression phenotype connected to FUS proteinopathy. We propose that the nuclear exclusion of Ipl1 through indirect interaction with FUS drives the dysregulation of H3S10ph as well as H3K14ac via crosstalk. We found that the knockdown of Nop1 interferes with these processes. In a parallel mechanism, Rtt109 mislocalization results in reduced levels of H3K56ac. Our results highlight the contribution of epigenetic mechanisms to ALS/FTD and identify novel targets for possible therapeutic intervention.

## 1. Introduction

Amyotrophic lateral sclerosis (ALS) is a devastating neurodegenerative disease characterized by the loss of upper and lower motor neurons [1]. Frontotemporal dementia (FTD) is characterized by the neurodegeneration of the frontal and temporal lobes of the brain. FTD is the leading cause of dementia in people under 60 [2]. The discovery of shared pathological and genetic features has established ALS and FTD to lie on two ends of a disease spectrum [3]. There is no cure for ALS/FTD, and the prognosis is poor [3,4,5]. While the true origin of ALS/FTD’s pathology remains unclear, a multitude of genes have been associated with ALS/FTD, including *Fused in Sarcoma* (*FUS*) [6].

FUS is an RNA-binding protein, primarily localized to the nucleus and involved in numerous cellular processes including RNA maturation and DNA repair [7,8]. In ALS/FTD, FUS mislocalizes to the cytoplasm and aggregates [9,10,11]. A multitude of *FUS* mutations have been observed in ALS patients; mutations in the highly conserved nuclear localization signal are pathogenic [7,12]. Similarly, FUS aggregates have been observed in FTD patients [7]. FUS aggregation has been associated with stress granule formation and the formation of prion-like aggregates, but its pathogenic mechanism is still poorly understood [7].

Epigenetics refers to heritable alterations in gene expression occurring without modifications to the underlying genome [13]. The main molecular epigenetic mechanisms include DNA methylation, microRNAs, and histone post-translational modifications (PTMs) [13]. Chromatin is made up of DNA wrapped around histone proteins. The core unit of chromatin is a nucleosome, which consists of 146 base pairs of DNA wrapped around a histone octamer (comprising two H2A/H2B dimers and an H3/H4 tetramer) [14]. The terminal tails of the histone proteins protrude from the nucleosome and are heavily modified with varied chemical moieties, including methylation, acetylation, phosphorylation, and ubiquitination [14]. Some of the enzymes responsible for the deposition and removal of these groups are histone acetyltransferases (HATs), histone deacetylases (HDACs), kinases, phosphatases, and histone methyltransferases [15,16,17,18]. Histone modifications comprise a ‘code’ that other proteins can ‘write’, ‘erase’, and ‘read’ [19]. Histone modifications can also display ‘cross-talk’, where one modification can promote the deposition or removal of another modification in the same or a different histone [20].

Recent work has linked several histone-modifying enzymes to ALS/FTD. For instance, the RNAi silencing of FUS reduced the expression of HDAC 6 mRNA [21]. Moreover, HDAC inhibition has arisen as a promising therapeutic in various ALS/FTD models. For example, motor neuron degeneration was ameliorated by treatment with Trichostatin A, an HDAC inhibitor, in a mouse model [22]. In transgenic ALS mice, HDAC inhibition with 4-PB increased motor function and neuroprotection [23]. In motor neurons derived from ALS patients, pharmacological inhibition and the genetic silencing of HDAC 6 led to neuroprotective effects by reversing axonal transport defects caused by mutant FUS as well as preserving DNA repair mechanisms [24,25,26]. These results suggest targeting histone-modifying enzymes as a possible therapeutic approach for ALS/FTD; however, little is known about the mechanisms linking protein aggregation to the epigenome.

Exploiting a FUS proteinopathy yeast model [27], we have previously shown that the levels of specific histone PTMs are significantly depleted in this context. In particular, the levels of H3S10ph, H3K14ac, and H3K56ac are decreased [27]. Furthermore, treatment with Trichostatin A bypasses FUS toxicity in yeast by restoring the levels of H3K14ac and H3K56ac without affecting FUS expression or aggregation [28]. This suggests that the detrimental effect of protein aggregation is at least partially related to its association with aberrant histone marks [28]. To expand on these findings, here we explore the connections between FUS proteinopathy and the histone PTM landscape in a yeast FUS overexpression model. First, we probed for the levels and cellular localization of the histone-modifying enzymes responsible for the deposition of histone PTMs linked to FUS proteinopathy. We interrogated Ipl1, Gcn5, and Rtt109, which are responsible for installing H3S10ph, H3K14ac, and H3K56ac, respectively [17,29,30,31]. We found that Ipl1 and Rtt109 are excluded from the nucleus, while Gcn5′s localization remains unchanged. Next, we cataloged the FUS yeast interactome by way of co-immunoprecipitation (Co-IP) coupled with mass spectrometry (MS)-based proteomics. Notably, we identified Nop1 and Rrp5 as FUS binding partners. Nop1 is an essential yeast histone methyltransferase required for the expression of pre-rRNA [32], while Rrp5 is an essential RNA-binding protein involved in the synthesis of 18S and 5.8S rRNAs [33]. Finally, we established Rrp5 and Nop1 as players in FUS’s cytotoxic effects in yeast, as lowering the abundance of these proteins through mRNA perturbation leads to the remarkable amelioration of growth suppression even in the context of robust FUS expression and aggregation. Interestingly, the knockdown of these proteins connects to different effects on the FUS-linked histone PTM alterations. Altogether, we present a model where Ipl1 and Rtt109 are mislocalized to the cytoplasm, causing a reduction in the levels of H3S10ph and H3K56ac, respectively. Our data suggests an indirect interaction between Ipl1 and FUS due to colocalization. We also show that Gcn5 does not mislocalize from the cytoplasm with respect to FUS overexpression, alluding to decreased H3K14ac levels being a result of histone crosstalk. Altogether, our results highlight potential openings for therapeutic intervention in ALS/FTD.

## 2. Materials and Methods

### 2.1. Yeast Strains, Media, and Plasmids

PCR targeting, co-immunoprecipitation, and mass spectrometry were performed with W303a yeast (*MATa*, *can1-100*, *his3-11,15*, *leu2,3,11,12*, *trp1-1*, *ura3-1*, *ade2-1*) [34]. Serial growth dilution assays, microscopy, and histone Western blots were performed with BY4741 yeast (*MATα his3Δ1 leu2Δ0 ura3Δ0 met15Δ0*) from the yeast DAmP library (Horizon Discovery, Cambridge, UK) [35]. Yeast was grown in a synthetic dropout medium (Clonetech Laboratories, Mountain View, CA, USA) supplemented with 2% glucose, raffinose, or galactose (BY4741 DAmP strains were additionally supplemented with 200 μg/mL G418 (ThermoScientific, Waltham, MA, USA; cat. no. 329400050)). The integrating FUS plasmid (pAG303GAL-FUS) was a gift from A. Gitler (Addgene plasmid no. 29614) [8]. The integrating TDP-43 plasmid (pAG303GAL-TDP-43) was a gift from M. Jackrel and J. Shorter [36]. A control integrating ccdB plasmid, pAG3030GAL-ccdB, was a gift from S. Lindquist (Addgene plasmid no. 14133) [34]. The yeast was transformed using standard poly(ethylene glycol) and lithium acetate protocols [27,37].

### 2.2. Transformation of Yeast with FLAG-Tagged Histone-Modifying Enzymes via PCR Targeting

We designed primers recognizing the FLAG and KanMX sequence in the pTF268 plasmid (Addgene plasmid no. 44095) at the 5′ and 3′ ends; they were created with 40 base overhangs recognizing the end of either Ipl1, Gcn5, or Rtt109 up to the stop codon (forward primer, Supplementary Table S1) and the immediate bases after the stop codon (reverse primer, Supplementary Table S1). The primers were then used in a PCR reaction (Phusion High-Fidelity PCR Kit, New England BioLabs, Ipswich, MA, USA, Cat. No. E0553L) using the pTF268 plasmid as a template to create a transforming insert. The inserts were then used to transform FUS and control yeast using a high-efficiency yeast transfer protocol [38]. We selected for transformed colonies by plating yeast on synthetic dropout media plates containing 450 μg/mL G418. The homologous recombination with the target gene was confirmed through PCR using yeast lysate as the template with a forward primer recognizing an area of the gene of interest and a reverse primer recognizing the FLAG sequence (Supplementary Table S1).

### 2.3. Protein Overexpression

Yeast strains were grown to saturation overnight in raffinose-supplemented dropout media (BY4741 DAmP strains were additionally supplemented with 200 μg/mL G418) at 30 °C and 200 rpm. Overnight cultures were then diluted to an OD_600_ of 0.30 in galactose-supplemented synthetic dropout media (BY4741 DAmP strains were additionally supplemented with 200 μg/mL G418) and induced for 5 h at 30 °C. Yeast cultures were then standardized to the lowest OD_600_. Cells were then pelleted at 850 rcf at 4 °C and washed 3X with sterile distilled water and harvested. The supernatant was removed, and the pellets were flash-frozen in liquid nitrogen and stored at −80 °C.

### 2.4. Serial Dilution Growth Assays

Yeast was grown to saturation overnight in raffinose-supplemented dropout media (BY4741 DAmP strains were additionally supplemented with 200 μg/mL G418) at 30 °C. Overnight cultures were diluted 2-fold, then serially diluted 5-fold. The yeast was spotted onto a synthetic dropout medium containing glucose or galactose (BY4741 DAmP strains were additionally supplemented with 200 μg/mL G418) with a pin-frogger. The yeast was grown at 30 °C for 3 to 4 days before imaging. Images of the plates were imported into ImageJ (Fiji version 2.9.0 ), and the density of the middle spot of each plate was measured using the oval tool. All experiments were repeated a minimum of three times with three independently transformed yeast strains.

### 2.5. Western Blotting

Western blotting was performed as previously described [27,37]. Briefly, frozen yeast cell pellets were thawed and treated with 0.2 M NaOH for 10 min on ice, pelleted again, and subsequently resuspended in 100 μL of a 1X SDS sample buffer and boiled for 10 min. Cell lysates were separated using SDS-PAGE and then transferred to a PVDF membrane (EMD Millipore, Burlington, MA, USA). The membranes were blocked using a LI-COR blocking buffer (LI-COR Biosciences, Lincoln, NE, USA) for 1 h at RT. The membranes were incubated with primary antibodies at 4 °C overnight. The primary antibodies used included rabbit anti-FUS polyclonal (Bethyl Laboratories, Montgomery, TX, USA; cat. no. A300-302A, 1:1000 dilution), rabbit anti-TDP-43 polyclonal (Proteintech, Rosemont, IL, USA; cat. no. 10782-2-AP, 1:1000 dilution), mouse anti-PGK1 monoclonal (Novex, Frederick, MD, USA; cat. no. 459250, 1:2000 dilution) (discontinued), mouse anti-PGK1 monoclonal (Abcam, Cambridge, MA, USA; cat no. ab113687, 1:2500 dilution), mouse anti-H3 total (Abcam, Cambridge, MA, USA; cat. no. ab24834, 1:2000 dilution), rabbit anti-H3S10ph (Abcam, Cambridge, MA, USA; cat. no. ab5176, 1:1000 dilution), rabbit anti-H3K14ac (Millipore, cat. no. 07-353, 1:2000 dilution), rabbit anti-H3K56ac (Active Motif, Carlsbad, CA, USA; cat. no. 39281, 1:5000 dilution), mouse anti-α-Tubulin (1:10,000), and mouse anti-Flag M2 antibodies (cat. no. F3165). Blots were processed using goat anti-mouse and anti-rabbit secondary antibodies from LI-COR Biosciences (both at a 1:20,000 dilution) and imaged using an Odyssey Fc imaging system (LI-COR Biosciences). All immunoblotting experiments were independently repeated a minimum of three times. The densitometric analysis of Western blots was performed using Image Studio (LI-COR Biosciences). The signals obtained for the histone modifications were normalized to their respective total H3 signals (modification/total H3). These values were then compared with untreated control/sample values to obtain relative density values (sample/control), which were used for statistical analysis. Similarly, FLAG bands were compared to α-Tubulin signal values for normalization.

### 2.6. Co-Immunoprecipitation

Control and FUS yeast were grown until an O.D. of ~0.8 and harvested through centrifugation at 850 rcf at 4 °C for 5 min, followed by two washes with sterile water. The resulting pellet was spheroplasted through resuspension in 500 μL of spheroplasting solution (1.2 M D-sorbitol; 0.5 mM MgCl_2_; 20 mM Tris—pH 7.5; 50 mM β-mercaptoethanol; 0.5 mg/mL Zymolyase-100T) with constant rotation at 30 °C for one hour, followed by harvesting at 800 rcf for 5 min at room temperature. The resulting pellet was then resuspended in 200 μL lysis buffer (20 mM Tris—pH 7.5; 10 mM β-mercaptoethanol; 0.5% Triton X-100; 2X HALT Protease Inhibitor) and incubated for 10 min at room temperature, followed by centrifugation at 4000 rcf for 5 min. The resulting supernatant was used immediately for Co-IP. FUS or Nop1 antibodies were conjugated to M270 Dynabeads and the co-immunoprecipitation was completed using the Dynabeads Co-Immunoprecipitation Kit according to the manufacturer’s specifications using the highest stringency washes (Invitrogen, Waltham, MA, USA, cat. no. 14321D). The resulting proteins were analyzed via Western blotting.

### 2.7. Tandem Mass Spectrometry

For samples analyzed through mass spectrometry, the bead–cell lysis slurry was washed four times with sterile filtered PBS and submitted for MS analysis (NYU Proteomics Core, West Tower, NY, USA). Briefly, proteins were digested on beads by reducing them with DTT at 57 °C for 1 h, followed by alkylation with Iodoacetamide at room temperature for 45 min and digestion with sequencing-grade Trypsin overnight. The samples were then de-salted using a C-18 membrane and washed three times with 0.1% TFA. The samples were then eluted with 0.5% acetic acid and dried in a SpeedVac. The samples were then reconstituted in 0.5% acetic acid and separated through LC using an EASY-nLC 1220 (ThermoScientific). Peptides were gradient-eluted from the column directly to an Orbitrap Eclipse (ThermoScientific) using a 1 h gradient (Solvent A: 2% acetonitrile, 0.5% acetic acid; Solvent B: 80% acetonitrile, 0.5% acetic acid). High-resolution full MS spectra were acquired with a resolution of 240,000, an AGC target of 1,000,000, a maximum ion time of 60 ms, and a scan range of 400 to 1500 *m*/*z*. All MS/MS spectra were collected using the following instrument parameters: in the ion trap, there was an AGC target of 2000, a maximum ion time of 18 ms, one microscan, a 2 *m*/*z* isolation window, a fixed first mass of 110 *m*/*z*, and an NCE of 27. MS/MS spectra were searched against the Uniprot Yeast database using Sequest within Proteome Discoverer 1.4 (ThermoScientific). The proteins of interest were determined by considering any protein that appeared in both trials, had a peptide spectral match over 40, and was at least enriched two-fold compared to the controls.

### 2.8. Immunocytochemistry

FUS and control yeast containing either Ipl1-FLAG, Gcn5-FLAG, or Rtt109-FLAG were imaged using a standard protocol [39]. Briefly, cells were fixed for 15 min at constant rotation in 1 mL 4% paraformaldehyde solution (Ted Pella, Reeding, CA, USA, cat. no. 18501; in 0.1 M sucrose), followed by 2 washes in 1 mL KPO_4_ and 1 wash with 0.1 M KPO_4_/1.2M sorbitol. The cells were then resuspended in 1 mL of 0.1 M KPO_4_. Yeast was then spheroplasted for 12~13 min in 0.1 M KPO_4_, 0.3 M β-mercaptoethanol, and 0.1 mg/mL Zymolase-100T, followed by two washes with 0.1 M KPO_4_, and was harvested through 1 min of centrifugation in a microcentrifuge. The cells were resuspended in 50 μL 0.1 M KPO_4_. A total of 15 μL of the cells were then adhered to Teflon-coated slides that were coated with 0.1% poly-lysine (Epredia, Portsmouth, NH, USA, cat. no. 86-010), and the supernatant aspirated off. The slide was immediately submerged into ice-cold methanol for 6 min, followed by submersion in room temperature acetone, and was quickly air-dried. The cells were then blocked for 30 min with 25 μL PBS-BSA (150 μM Bovine Serum Albumin, 0.05 M KPO_4_, 0.15 M NaCl, 30 mM NaN_3_). The cells were then incubated with a primary antibody overnight (1:400 for FUS, 1:100 for FLAG), followed by 5 washes with a blocking buffer, 1.25 h incubation with a secondary antibody (anti-rabbit AlexaFluor-488, 1:500, Life Technologies, Carlsbad, CA, USA, cat. no. A11008; anti-mouse AlexaFluor-586, 1:1000, Life Technologies, Carlsbad, CA, USA, cat. no. A11004), 5 washes with a blocking buffer, and finally 2 washes with sterile filtered PBS. All the volumes were 25 μL/well and all steps after the addition of the secondary antibody took place in the dark. The cells were mounted with 5 μL Fluoromount-G Mounting Medium with DAPI (Invitrogen, Waltham, MA, cat. no. 00-4959-52). The slides were imaged on a Zeiss LSM 800 confocal microscope at a 63× magnification using the DAPI, AF488, and AF555 lasers. The laser intensity was kept constant between the control and FUS samples. The resulting images were processed using ImageJ [40]. The nuclear intensity was calculated from each image by thresholding the DAPI image until only the nucleus was in view, selecting the area and superimposing it over the FLAG channel, and measuring the mean fluorescence intensity and area. The whole-cell FLAG intensity was measured by thresholding the FLAG image until only the cells were in view, selecting the area, and measuring it. The cytoplasmic intensity was then calculated using the formula [41]

*Cytoplasmic Intensity* = (*Whole Cell Mean* × *Whole Cell Area*) − (*Nuclear Mean* × *Nuclear Area*)

and the percent of nuclear localization was calculated using the formula [41]

%*Nuclear* = *Nuclear Intensity*/(*Cytoplasmic Intensity* + *Nuclear Intensity*)

### 2.9. Statistical Analysis

The statistical analysis of the data was performed using GraphPad Prism ver. 10.0.0 (GraphPad Software, Boston, MA, USA). Significant differences between the nuclear intensity, histone-modifying enzymes, and PTM levels were determined using Welch’s *t*-test, with *p* = 0.05 as the cutoff. Error bars on the graphs represent the standard deviation (SD) calculated from the values obtained in the data analysis steps described above. All data were analyzed with the Robust Regression Outlier Test (ROUT) to identify outliers with a Q = 2% [42]. All graphs were constructed with GraphPad Prism ver.10.0.0 (GraphPad Software, Boston, MA, USA) [43].

## 3. Results and Discussion

### 3.1. Levels of Histone-Modifying Enzymes Are Not Decreased in Yeast Overexpressing FUS

FUS proteinopathy is connected to changes in the levels of H3S10ph, H3K14ac, and H3K56ac [27]. FUS overexpression in yeast also leads to a decrease in the total RNA levels [27]. Furthermore, histone PTM dysregulation linked to FUS overexpression occurs independently of FUS’ RNA-binding ability [44]. Decreased histone PTM levels potentially suggest decreases in the levels of the histone-modifying enzymes (HMEs) responsible for each respective PTM. Hence, we focused on the writers of these histone PTMs. H3S10ph, H3K14ac, and H3K56ac are installed by the enzymes Ipl1, Gcn5, and Rtt109, respectively. Interestingly, the inhibition of Aurora B kinase, the human homolog of Ipl1, has been shown to increase mitochondrial transport in the axon of motor neurons derived from ALS patients [45,46]. Mammals have two homologs of the histone acetyltransferase (HAT) Gcn5: GCN5L2 and p300/CBP [47]. The loss of p300 and decreased histone acetylation have been associated with ALS [48]. In the case of Rtt109, structural data have shown that its metazoan homolog is p300/CBP [49]. Unlike yeast, in humans, H3K56 is not acetylated by a single HAT but by a combination of GCN5L2 and p300/CBP [50].

Using a W303 yeast strain bearing a FUS plasmid (pAG303GAL-FUS) with a galactose-inducible promoter, we set out to determine if the levels of relevant histone modifiers were impacted by FUS overexpression [27,28]. Unfortunately, we could not directly assess the levels of Ipl1, Gcn5, and Rtt109 via immunoblotting because there are no commercially available antibodies for these enzymes in yeast. To circumvent this problem, we attached a FLAG tag to the C-termini of the Ipl1, Gcn5, and Rtt109 genes via PCR targeting [38]. Inserts were created with primers containing part of the genomic sequence of each enzyme and the 3X Flag—KanMX region of the pTF270 plasmid (Supplementary Table S1 and Figure S1a). The resulting DNA inserts were transformed into yeast already possessing integrated control and FUS plasmids. The insertion of the FLAG tag was verified by selection against G418, as well as PCR using forward primers recognizing the gene of interest and a universal primer recognizing the FLAG region of the insert (Supplementary Figure S1b).

**Figure 1:**
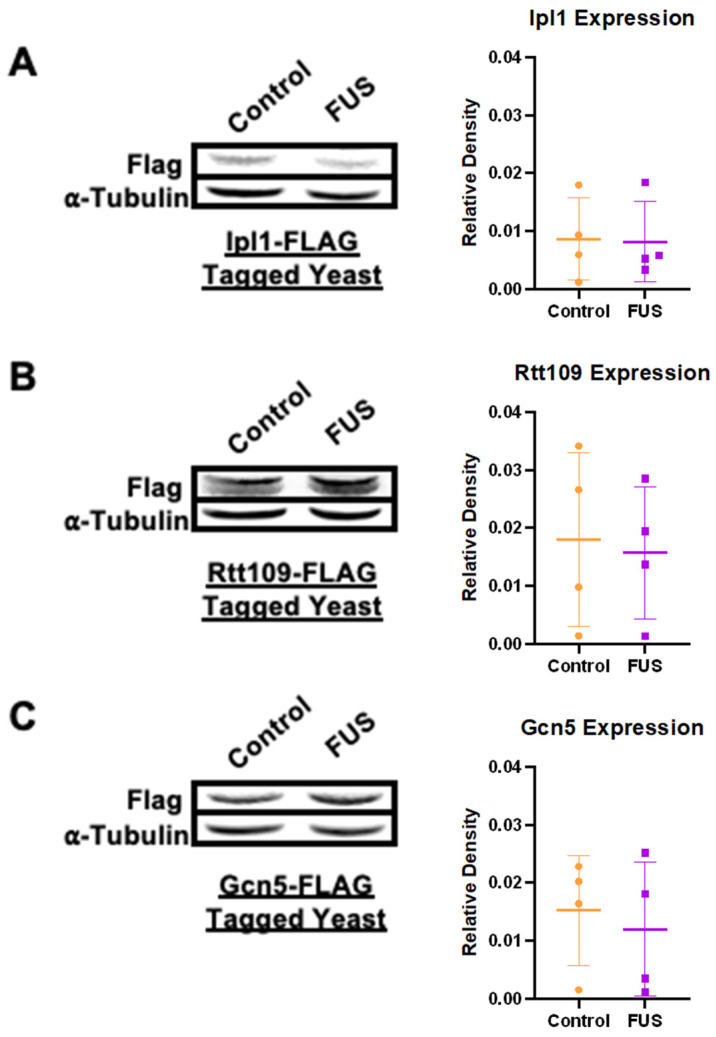
**Levels of select histone-modifying enzymes remain unchanged in connection to FUS proteinopathy.** The levels of (**A**) Ipl1, (**B**) Rtt109, and (**C**) Gcn5 were measured through immunoblotting against FLAG in yeast overexpressing a control (orange) or FUS (purple) vector. α-Tubulin was used as a loading control. Column scatterplots compiling multiple independent biological replicates display the mean fold change in the FLAG expression based on densitometric analysis. Error bars represent ±SD. *n* = 4.

Exploiting this setup, we measured the overall expression levels of FLAG-tagged Ipl1, Gcn5, and Rtt109 in yeast overexpressing FUS and a vector control via immunoblotting. The levels of FLAG-tagged enzymes measured through densitometric analysis were standardized to the Tubulin levels in each strain. Surprisingly, we did not observe any differences in the levels of HMEs between FUS and control yeast (Figure 1). These results support the assertion that the reduction in the H3S10ph, H3K14ac, and H3K56ac levels was not tied to a reduction in the levels of the enzymes responsible for their deposition. The lack of a reduction in histone PTM writers suggests alternative mechanisms for PTM reduction in FUS proteinopathy.

### 3.2. Ipl1-FLAG Colocalizes with FUS and Is Depleted from the Nucleus

**Figure 2:**
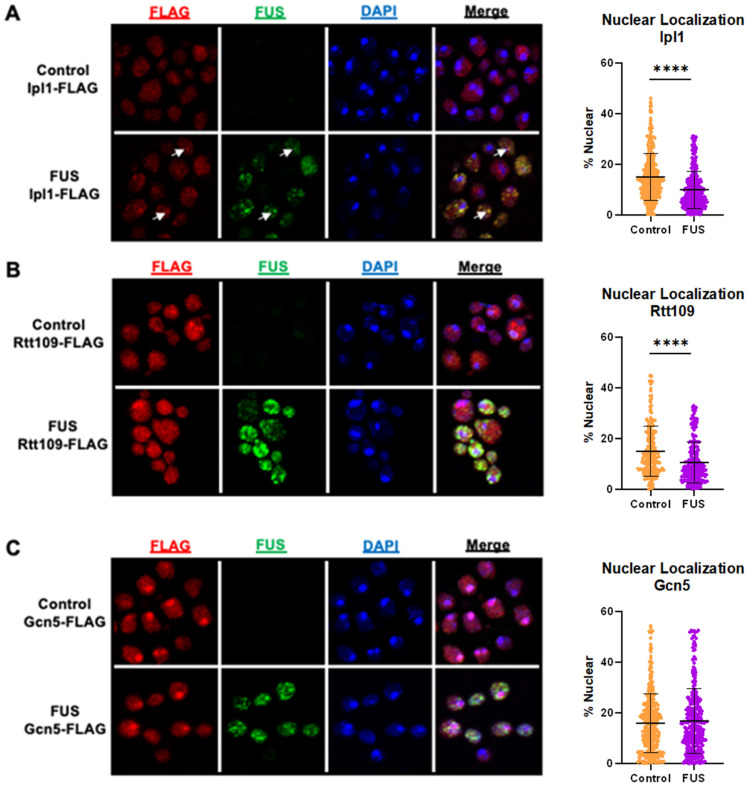
**Ipl1 and Rtt109 are depleted from the nucleus in FUS proteinopathy yeast models.** FUS or control yeast expressing (**A**) Ipl1-FLAG (*n* = 368 controls, 360 FUS), (**B**) Rtt109-FLAG (*n* = 199 controls, 235 FUS), or (**C**) Gcn5-FLAG (*n* = 348 controls, 315 FUS) were imaged using immunofluorescence with antibodies recognizing FLAG (red) and FUS (green) and counterstained with DAPI (blue). Column scatterplots represent the percent of the FLAG signal in the nucleus. Examples of Ipl1-FLAG and FUS colocalization are highlighted with white arrows. **** = *p* < 0.0001.

As the total levels of Ipl1, Gcn5, and Rtt109 were not impacted by FUS proteinopathy (Figure 1), we then explored whether their cellular localization was altered in this context. We performed immunocytochemistry in FUS and control yeast expressing Ipl1-FLAG, Gcn5-FLAG, or Rtt109-FLAG. From microscopy data, we then calculated the percent of nuclear localization for FLAG-tagged proteins [41]. In the case of Ipl1-FLAG, there was diffuse FLAG staining throughout the cells with distinct puncta in both control and FUS yeast (Figure 2A, left column). As expected, we did not detect any FUS signals in control yeast, whereas FUS yeast displayed robust staining in the cytoplasm (Figure 2A, second column from the left). Notably, the percentage of nuclear Ipl1 was lowered in FUS yeast (approximately 10%) compared to the control (approximately 15%) (Figure 2A, column scatter plot), suggesting that decreased H3S10ph levels may be caused by the exclusion of Ipl1 from the nucleus. We also observed numerous yellow puncta in FUS Ipl1-FLAG yeast, suggesting colocalization between the two fluorophores (Figure 2A, fourth column from the left, white arrows). These results suggest that decreased levels of H3S10ph are related to the cellular mislocalization of Ipl1 in FUS yeast. The colocalization between the two proteins suggests that Ipl1 interacts either directly or indirectly with FUS and that this interaction partially consigns Ipl1 to the cytoplasm and prevents Ipl1 from phosphorylating histone H3. A similar mechanism has been observed between FUS and the arginine methyltransferase PRMT1, in which FUS forces PRMT1 out of the nucleus, leading to reduced levels of histone methylation and acetylation [51].

### 3.3. Rtt109 Is Depleted from the Nucleus in Yeast Overexpressing FUS

We imaged FUS and control yeast expressing Rtt109-FLAG (Figure 2B). There was diffuse FLAG staining throughout the cells with distinct puncta in both control and FUS yeast (Figure 2B, left column). As before, we did not detect any staining for FUS in the control yeast, whereas FUS yeast displayed robust staining in the cytoplasm (Figure 2B, second column from the left). Remarkably, the percentage of nuclear FLAG staining in FUS yeast was approximately 11%, a modest decrease compared to the control cells with 15% nuclear FLAG staining (Figure 2B, column scatterplot), suggesting that lowered H3K56ac levels may be related to the nuclear exclusion of Rtt109. However, we did not observe colocalization of Rtt109-FLAG and FUS (Figure 2B, fourth column from the left). The absence of colocalization suggests that the redistribution of Rtt109-FLAG is not brought about by interactions between FUS and Rtt109. From these data, we were unable to determine the molecular events triggering Rtt109’s exclusion from the nucleus. Interestingly, FUS has been shown to block nucleocytoplasmic transport and may be eliciting Rtt109 mislocalization through reduced nuclear import [52,53]. The presence of H3K56ac is a sign of new histone H3 incorporation into chromatin after DNA damage repair [54,55]. Interestingly, this mark is also important for the expression of rRNA [56,57]. The H3K56ac loss may be contributing to the reduced levels of total RNA we have observed in yeast overexpressing FUS [27]. While Rtt109’s nuclear exclusion implicates its dysregulation in FUS proteinopathy, further investigation is needed to establish such a mechanism.

### 3.4. Gcn5 Localization Is Unchanged in Yeast Overexpressing FUS

As for Ipl1 and Rtt109, we probed for the localization of Gcn5-FLAG in yeast overexpressing FUS or a control vector (Figure 2C). There was diffuse FLAG staining throughout the cells with distinct puncta in both control and FUS yeast (Figure 2C, first column from the left). Expectedly, we did not see any staining for FUS in control yeast, whereas FUS yeast displayed robust staining in the cytoplasm (Figure 2C, second column from the left). We did not observe any difference in the amount of nuclear FLAG staining between control and FUS yeast (approximately 16% for each), suggesting Gcn5 is not redistributed in connection with FUS proteinopathy (Figure 2C, column scatterplot). Similarly, we did not observe colocalization between Gcn5-FLAG and FUS (Figure 2C, fourth column from the left). As Gcn5 did not mislocalize to the cytoplasm or colocalize with FUS, we postulate that the reduction in H3K14ac levels might occur via histone crosstalk. In fact, H3S10ph is known to drive H3K14ac in yeast, so reductions in the levels of H3S10ph could lead to decreases in the levels of H3K14ac [58]. Alternatively, it is possible that the increased activity or aberrant localization of Rpd3, the HDAC responsible for removing H3K14ac [59], was driving the decrease in H3K14ac levels.

### 3.5. Putative Binding Partners of FUS Are Involved in ATP Binding and rRNA Processing

**Figure 3:**
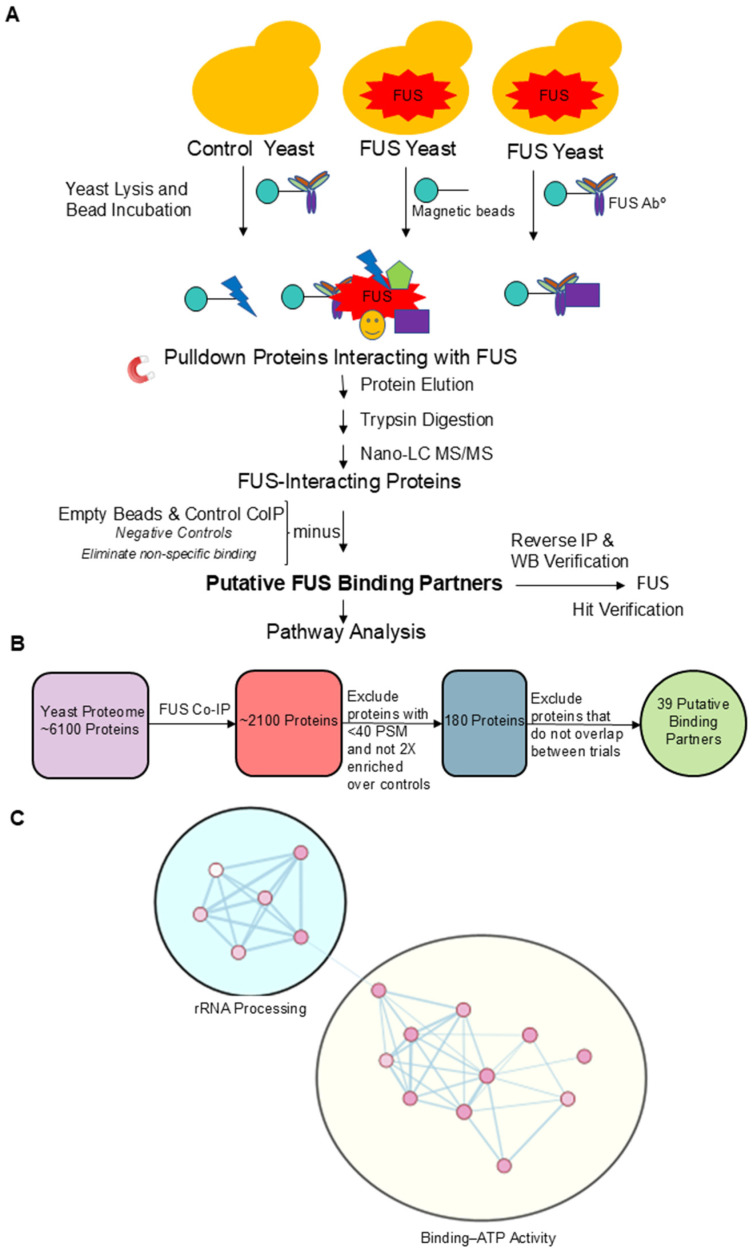
**Putative binding partners of FUS are involved in rRNA processing and ATP binding.** (**A**) Schematic representation of co-immunoprecipitation experiments using an FUS antibody as bait. Negative controls are also shown. (**B**) Diagram portraying filtering of FUS Co-IP protein hits. (**C**) Enrichment map created from GO annotations and KEGG Pathways associated with putative FUS yeast binding partners. Nodes highlighted in the yellow oval correspond to annotations related to ATP binding, and nodes highlighted in the teal circle correspond to annotations involved in rRNA processing.

To gain insight into the molecular connections between protein aggregation and the epigenome, we interrogated FUS’s protein interactome in yeast by performing a co-immunoprecipitation experiment (Co-IP) using an FUS antibody as bait (Figure 3A). Yeast does not have a homolog for FUS; therefore, genetic interference from the native protein is not an issue. We covalently attached a rabbit polyclonal FUS antibody to magnetic beads and incubated these beads with lysates from W303 yeast overexpressing FUS. As a negative control, we incubated beads conjugated to the FUS antibody with lysates from yeast overexpressing a vector control (pAG3030GAL-ccdB; no FUS). This control accounted for non-specific protein binding to the FUS antibody. As an additional negative control, we also incubated non-conjugated “naked” beads with FUS yeast lysates to exclude those proteins binding non-specifically to the beads themselves. The recovered proteins were separated using SDS-PAGE and visualized through silver staining (Supplementary Figure S2a). There were several distinct protein bands in the FUS lane. In the FUS sample, there was a prominent band near the 34 kDa molecular weight marker. Another band between the 43 and 55 kDa molecular weight markers was also present. A strong band around 55 kDa was likely FUS itself. We confirmed FUS pulldown via Western blotting (Supplementary Figure S2b).

To identify FUS’s binding partners, recovered proteins from two independent Co-IP experiments were analyzed using tandem mass spectrometry. Each trial identified over 2000 proteins as interacting with FUS. To narrow down the proteins of interest, we only considered proteins that (1) had over 40 peptide spectrum matches in the FUS Co-IP condition, (2) displayed at least two-fold enrichment over the two control conditions, and (3) were among the hits in both trials. A peptide spectrum match refers to the number of times a peptide is identified and relates to the abundance of a particular protein in the sample [60]. This filtering process rendered 39 proteins as putative FUS binding partners (Figure 3B; Supplementary Table S2). Notably, we did not observe Ipl1 or Rtt109 as putative binding partners of FUS, even though Ipl1 colocalizes with FUS, suggesting that Ipl1′s sequestration in the cytoplasm is not due to a direct interaction between these proteins.

To better understand what cellular functions FUS proteinopathy impacts, we submitted our final 39 proteins for pathway analysis with the online tool DAVID [61,62]. The resulting Gene Ontology and KEGG Pathway annotations are shown in Supplementary Table S3 [63,64,65,66]. The top GO biological pathway and molecular function annotations are displayed in Supplementary Figure S3. Three of the four top GO biological annotations were involved in rRNA processing, while the top three molecular function annotations were involved in ATP binding. Interestingly, a large number of ribosomal proteins were identified as FUS partners in a GST-tagged FUS Co-IP study in 293T cells [67]. Using the resulting annotations, we created an enrichment map (Figure 3C). The nodes in the map represented annotations that were significantly over-represented among our 39 putative binding proteins, and the nodes were automatically clustered into similar biological processes. Out of 17 nodes, 11 were related to ATP binding (Figure 3C, yellow oval). Interestingly, many of the nodes that corresponded to ATP binding were related to ATP-dependent RNA helicase activity. Furthermore, six putative binding proteins (Dbp1, Dbp2, Ded1, Mcm4, Prp43, and Ylr419W) were RNA helicases [68,69,70,71,72]. Unsurprisingly, we also found the ATP-binding subunits of Hsp70 chaperones (Ssa1 and Ssa2) among our protein hits [73]. FUS is found in cytoplasmic aggregates (Figure 2), and Hsp70 is likely recruited to process such aggregates. Hsp70 expression is reduced in FUS-mutant motor neurons, and this reduction cannot be overcome by the chemical induction of Hsp70. The revelation that Hsp70 is a putative binding partner of FUS in yeast further suggests the dysregulation of heat shock pathways playing a role in FUS proteinopathy [25]. Hsp70 and Hsp104 are directly involved in protein disaggregation in yeast [74]. Intriguingly, Ju et al. revealed that the deletion of Hsp104 did not modify the toxicity of FUS in yeast, nor its aggregation or localization [75]. Ultimately, this suggests that while these disaggregases may bind to FUS, they may not be enough to fully break down these protein aggregates. Furthermore, the translational elongation factor Tef1 was also among our hits [76]. Yeast Tef1 is homologous to the eukaryotic elongation factor EEF1A2 found in humans, associated with neurodegeneration and proteostasis [77]. Interestingly, Tef1 has a molecular weight of 50 kDa and corresponds to the strong band observed in the silver staining of the FUS Co-IP between 43 and 55 kDa (Supplementary Figure S2b). FUS proteinopathy is associated with decreased gene expression [27], and Tef1 binding by FUS and nuclear exclusion might contribute to this. Tef1 is also essential for tRNA export in yeast, potentially implicating FUS proteinopathy in errors in non-coding RNA function [78,79]. The other six nodes were associated with rRNA processing (Figure 3C, teal oval). Five putative FUS binding proteins were in each of these six nodes: Utp10, Nop56, Rrp5, Utp22, and Nop1. Utp10 and Utp22 are both members of the small subunit processome, with the former being involved in the processing of pre-18SrRNA and the latter being necessary for the export of tRNAs from the nucleus [80,81]. Nop56 and Nop1 are essential nucleolar proteins involved in the methylation of pre-rRNA [82]. Lastly, Rrp5 is an essential RNA-binding protein involved in 18S and 5.85S rRNA biogenesis [33]. Gawade et al. identified that FUS KO HEK293T and SH-SY5Y models displayed a considerable hypermodification of rRNA species. This agrees with our data finding that several rRNA processing proteins bind to FUS, and taken together, this suggests that FUS plays a role in regulating the proper folding and translation efficiency of ribosomes [83]. The most represented FUS binding partners are all involved in yeast RNA processing, which is necessary for cellular health, providing another potential mechanism behind FUS-induced neurodegeneration [84].

### 3.6. Decreased mRNA of Rrp5 Alleviates FUS-Linked Growth Suppression but Does Not Impact Histone PTM Changes

**Figure 4:**
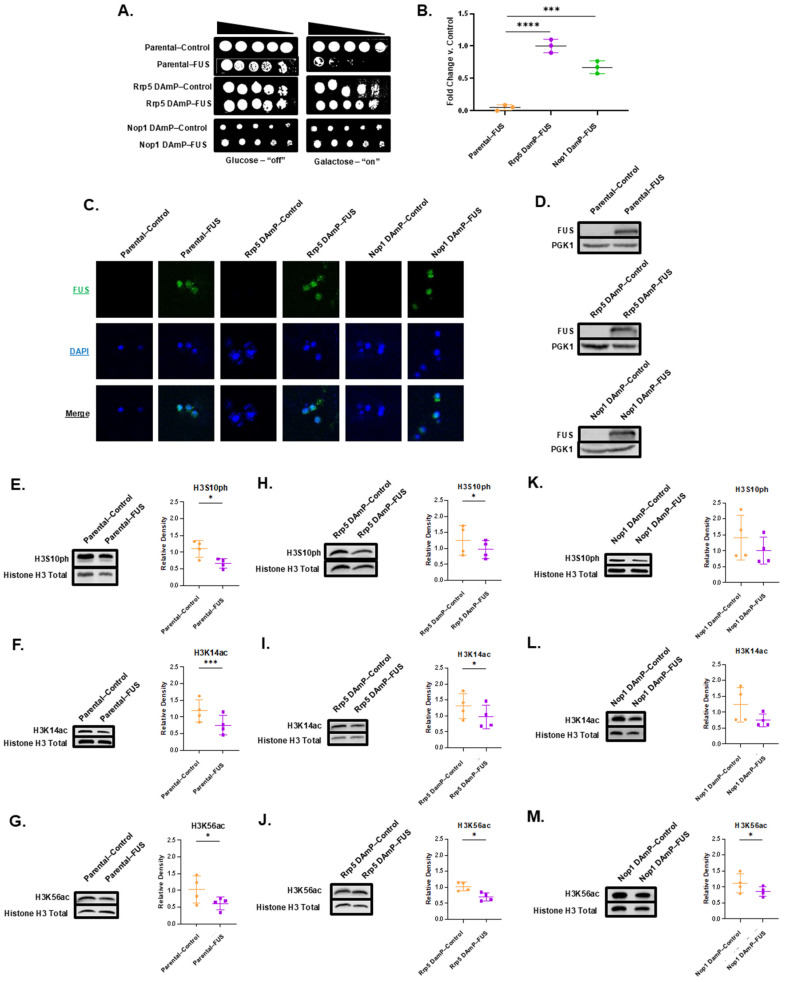
**Reduced levels of either Rrp5 or Nop1 mRNA relieve growth suppression but show differential effects on histone PTM levels in FUS-overexpressing yeast.** (**A**) Serial growth dilution assays depicted cell viability of parental, Rrp5 DAmP, and Nop1 DAmP control and FUS overexpression lines spotted on glucose (FUS “off”) or galactose (FUS “on”) media (*n* = 3). (**B**) Column scatterplot represents densitometric measurement of cell density of FUS yeast (middle spot) compared to control yeast on galactose plates in (**A**). *** = *p* < 0.001; **** = *p* < 0.0001. (**C**) Parental, Rrp5 DAmP, and Nop1 DAmP FUS or control yeast were imaged using immunofluorescence with antibodies against FUS (green) and counterstained with DAPI (blue). (**D**) Western blots confirmed the expression of FUS in these cells. *n* = 3. The levels of (**E**) H3S10ph, (**F**) H3K14ac, and (**G**) H3K56ac were measured in control (orange) and FUS (purple) parental yeast through immunoblotting. Similarly, levels of (**H**) H3S10ph, (**I**) H3K14ac, and (**J**) H3K56ac were measured in Rrp5 DAmP control and FUS yeast. Finally, levels of (**K**) H3S10ph, (**L**) H3K14ac, and (**M**) H3K56ac were measured in Nop1 DAmP control and FUS yeast. Column scatterplots compiling multiple biological replicates display the densities of histone post-translational modifications relative to the density of histone H3 as a loading control. Error bars represent ±SD. *n* = 4. * = *p* < 0.05; *** = *p* < 0.001.

**Figure 5:**
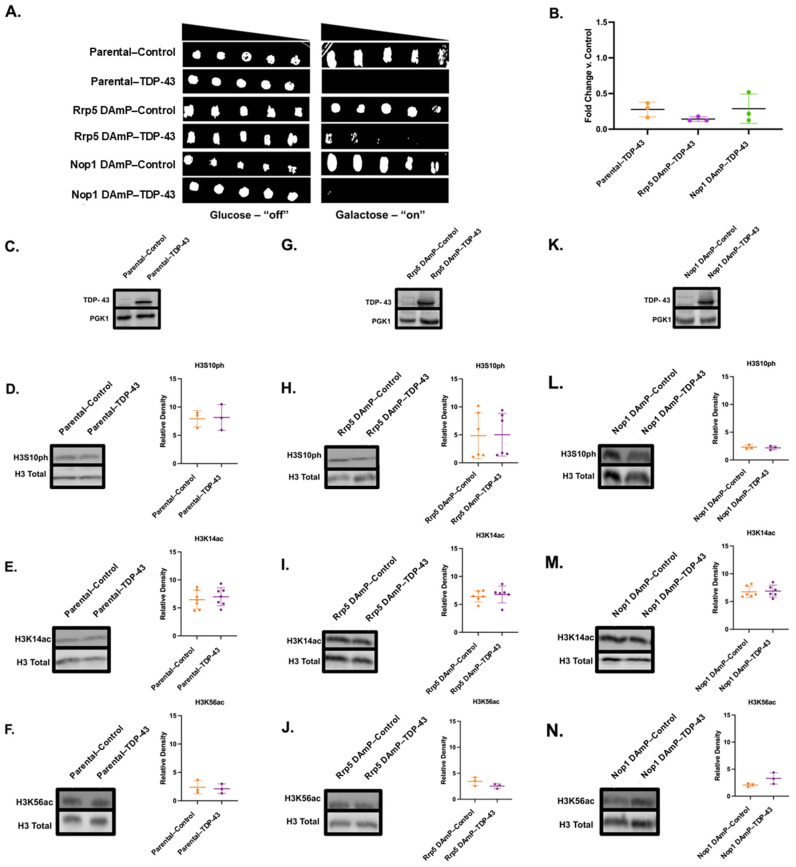
**Reduced Rrp5 or Nop1 mRNA levels do not affect TDP-43 overexpression levels, growth suppression, or histone PTMs.** (**A**) Serial growth dilution assays depicted cell viability of parental, Rrp5 DAmP, and Nop1 DAmP control and TDP-43 overexpression lines spotted on glucose (TDP-43 “off”) or galactose (TDP-43 “on”) media (*n* = 3). (**B**) Column scatterplot represents densitometric measurement of cell density of TDP-43 yeast (middle spot) compared to control yeast on galactose plates in (**A**). (**C**) Western blots confirm the expression of TDP-43 in parental cells. The levels of (**D**) H3S10ph, (**E**) H3K14ac, and (**F**) H3K56ac were measured in control (orange) and TDP-43 (purple) parental yeast through immunoblotting. Similarly, (**G**) expression of TDP-43 as well as levels of (**H**) H3S10ph, (**I**) H3K14ac, and (**J**) H3K56ac were measured in Rrp5 DAmP control and TDP-43 yeast. Finally, (**K**) expression of TDP-43 and levels of (**L**) H3S10ph, (**M**) H3K14ac, and (**N**) H3K56ac were measured in Nop1 DAmP control and TDP-43 yeast. Column scatterplots compiling multiple biological replicates display the densities of histone post-translational modifications relative to the density of histone H3 as a loading control. Error bars represent ±SD. *n* = 3–7.

We wanted to further understand whether FUS binding partners played a role in connecting FUS aggregation to changes to the histone post-translational landscape. Interestingly, Rrp5, an RNA-binding protein among our FUS binding partner results, has been found to associate with Ipl1 [85,86]. Rrp5 also has a human homolog, PDCD11, that enables NF-kappaB binding activity and is required for rRNA maturation and the generation of 18S rRNA [87]. Directly investigating this interaction through reverse co-immunoprecipitation was complicated by the fact that there is no commercially available Rrp5 antibody. Nevertheless, to establish if Rrp5 was involved in FUS’s toxicity and connection to the epigenome, we obtained a yeast strain from the yeast Decreased Abundance by mRNA Perturbation (DAmP) collection displaying reduced Rrp5 mRNA levels. DAmP strains allow us to explore perturbations to the expression levels of essential genes that cannot be completely knocked out [88]. The yeast DAmP library allows for reduced mRNA levels through the disruption of the 3′ UTR of the gene of interest with a kanamycin resistance cassette [88]. DAmP strains are available in a BY4741 background. To serve as a control, we also obtained a parental BY4741 line (dubbed “Parental” henceforth). Both strains were transformed to overexpress FUS. The overexpression of FUS was confirmed in each strain through immunofluorescence staining and Western blotting (Figure 4C,D). Overall, FUS overexpression levels were comparable in both the parental and Rrp5 DAmP strains. Serial dilution growth assays revealed that the parental strain recapitulated the growth suppression elicited by FUS overexpression in W303 yeast (Figure 4A,B) [27]. We verified that parental and DAmP strains bearing FUS constructs grew well in glucose-supplemented media (Figure 4A; Supplementary Figure S4). Intriguingly, the overexpression of FUS in Rrp5 DAmP yeast led to the relief of growth suppression (Figure 4A,B). These data suggest that Rrp5 is involved in the pathway linking FUS overexpression to cellular toxicity. To verify that Rrp5′s involvement in toxicity was specific to FUS proteinopathy, we overexpressed TDP-43—another protein whose aggregation is involved in ALS/FTD—in parental and Rrp5 DAmP yeast. We also verified that parental and DAmP strains bearing TDP-43 constructs grew well in glucose-supplemented media (Figure 5A; Supplementary Figure S5). Underscoring the specificity of Rrp5 knockdown in rescuing FUS overexpression and not general protein aggregation, we found that Rrp5 knockdown did not rescue growth suppression elicited by TDP-43 overexpression (Figure 5A,B).

Noting that FUS’s cytotoxic effects were alleviated by Rrp5 DAmP, we then wondered whether changes to the histone post-translational landscape would also be eliminated. We probed for changes in the levels of H3S10ph, H3K14ac, and H3K56ac in both parental and Rrp5 DAmP strains (Figure 4E–J). Previously, we have demonstrated that FUS overexpression in W303 yeast leads to an approximate 50% decrease in the genome-wide levels of H3S10ph, H3K14ac, and H3K56ac [27]. Recapitulating this finding, the parental BY4741 line overexpressing FUS exhibited an approximate 40% decrease in the levels of H3S10ph, H3K14ac, and H3K56ac, showing that these PTM changes are connected to FUS proteinopathy regardless of the yeast strain (Figure 4E–G). Parental BY4741 yeast overexpressing TDP-43 revealed no changes to the H3S10ph, H3K14ac, and H3K56ac levels, further exhibiting that these histone PTM changes are FUS-specific (Figure 5D–F). Remarkably, Rrp5 DAmP yeast overexpressing FUS revealed similar decreases in the levels of H3S10ph, H3K14ac, and H3K56ac compared to parental yeast. Specifically, H3K14ac levels dropped approximately 30% (Figure 4I), while H3S10ph and H3K56ac levels dropped about 25%, in cells overexpressing FUS compared to controls (Figure 4H,J). Similarly, Rrp5 knockdown yeast overexpressing TDP-43 showed no changes in the H3S10ph, H3K14ac, or H3K56ac levels (Figure 5H–J). Ultimately, Rrp5 knockdown yeast maintained decreased histone PTM levels in the context of FUS overexpression but displayed an alleviation of FUS’s growth suppression. Taken together, this suggests that FUS may lead to growth suppression and dysregulation in the histone PTM levels through two separate pathways. Alternatively, histone PTM alterations might be occurring upstream of Rrp5′s involvement in growth suppression.

The work presented here suggests the existence of two toxicity mechanisms triggered by FUS overexpression in yeast: (1) the augmentation of histone post-translational modification levels by altering the histone-modifying enzyme localization and (2) cellular toxicity promoted by the sequestration of several rRNA processing and ATP-binding proteins. Specifically, Rrp5 seems to connect to FUS toxicity independently of the epigenome. This raises the question, how do reduced levels of Rrp5 mRNA lead to an amelioration in growth suppression? The previous literature has identified a feedback loop between the regulation of rRNA transcription and the growth rate in *E. coli* and in yeast [89,90]. FUS overexpression may dysregulate this feedback loop, leading to the growth suppression noted in our parental strains. The knockdown of rRNA processing proteins such as Rrp5 may impact the rRNA–ribosome feedback system, leading to an overall overproduction of rRNA by other proteins that may remain in the nucleus. Hypothetically, this increase in the rRNA levels may stimulate cell growth in yeast, allowing cells to overcome the growth suppression elicited by FUS overexpression. However, further experiments would be needed to confirm this hypothesis.

### 3.7. Nop1 DAmP Alleviates Growth Suppression and Restores H3S10ph and H3K14ac Levels

Among FUS’s putative partners, we also found Nop1, the yeast homolog of fibrillarin, a protein involved in rRNA methylation, ribosomal small subunit biogenesis, and snoRNA localization, while also serving as an H2AQ104 methyltransferase [91]. Nop1 is involved in the transcription of rRNA [82]. Nop1 is also a histone-modifying enzyme responsible for the methylation of H2AQ105, a histone mark linked to the recruitment of RNA Polymerase I and rRNA biogenesis [92]. We were interested in Nop1 as it is a histone-modifying enzyme and thus relates more directly to the histone PTM landscape [81,93]. Additionally, fibrillarin has been reported to phase separate with FUS [94,95]. Just as with the parental and Rrp5 DAmP lines, Nop1 DAmP cells were transformed with either control or FUS overexpression plasmids. FUS overexpression was confirmed through immunofluorescence and Western blotting (Figure 4C,D). Akin to Rrp5 knockdown, a serial growth dilution assay of control and FUS-overexpressing Nop1 DAmP cells revealed that Nop1 knockdown rescues FUS’s growth suppression (Figure 4A,B). As for Rrp5, we verified this effect was specific to FUS proteinopathy by overexpressing TDP-43 in BY4741 and Nop1 DAmP yeast. We found that Nop1 knockdown does not rescue TDP-43 toxicity (Figure 5A,B). This finding emphasizes the specificity of Nop1 knockdown in rescuing FUS overexpression.

To explore the role of Nop1 in connecting FUS to the epigenome, we probed for the levels of H3S10ph, H3K14ac, and H3K56ac in Nop1 DAmP cells overexpressing FUS or a control vector. In contrast to Rrp5, Nop1 knockdown rescued changes in the levels of H3S10ph and H3K14ac (Figure 4K,L). Meanwhile, Nop1 DAmP FUS yeast showed a 20% decrease in H3K56ac levels compared to controls (Figure 4M). Much like parental and Rrp5 DAmP strains, Nop1 DAmP yeast overexpressing TDP-43 revealed no changes to any probed PTM (Figure 5L–N). These data suggest that Nop1 may be involved in the pathway causing PTM dysregulation, an alternative pathway to the potential Rrp5-mediated toxicity. Moreover, the lack of changes due to TDP-43 overexpression further supports the fact that these changes are specific to FUS proteinopathy and occur via interactions with FUS. Nop1 may be involved in the processes leading to reduced levels of nuclear Ipl1, resulting in decreased H3S10ph levels and reduced H3K14ac levels via crosstalk. Furthermore, the methylation of H2AQ105, installed by Nop1, is dependent on H3K56ac, and a reduction in H2AQ105me is linked to Rtt109 suppression [96]. Additionally, H3K56ac and H2AQ105me are both involved in recruiting small subunit processomes, two of which were revealed to be putative FUS binding partners, Utp10 and Utp22. Therefore, Rtt109 mislocalization may play a role in Nop1′s interaction with FUS, leading to crosstalk between H3K56ac and H2AQ105me [96]. Unfortunately, we were not able to measure the levels of H2AQ105me in yeast overexpressing FUS as there is no commercially available antibody for this modification. The dysregulation of H3K56ac levels and FUS-Nop1 binding suggest that decreased rRNA biogenesis could be contributing to FUS toxicity in yeast models. Supporting this hypothesis, FUS has been implicated in the response to DNA damage caused by topoisomerase-I. FUS localizes to sites of stalled RNA Polymerase I to modulate rRNA biogenesis [97]. Moreover, fibroblasts harvested from FUS ALS patients are hypersensitive to topoisomerase-I DNA damage, also suggesting a role for rRNA biogenesis in ALS [97]. Further investigations, such as exploring RNA polymerase activity and utilizing RNA probes, would be necessary to establish this hypothesis [98].

**Figure 6:**
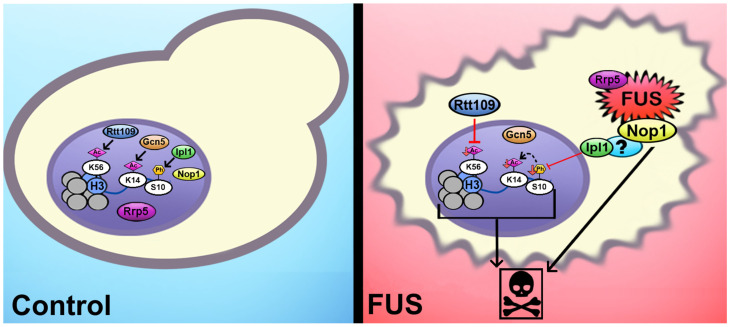
**Putative mechanisms linking histone PTMs to FUS proteinopathy in yeast.** Ipl1 is excluded from the nucleus through an indirect interaction with FUS, leading to reduced levels of H3S10ph. The H3K14ac levels are likely lowered through histone crosstalk with H3S10ph. A direct interaction between FUS and either Rrp5 or Nop1 is linked to cytotoxicity, while FUS’s interaction with Nop1 connects to changes in H3S10ph and H3K14ac. In a parallel mechanism, Rtt109 mislocalization contributes to the decrease in H3K56ac levels. All these associations do not occur in the context of TDP-43 proteinopathy and hence are not related to protein aggregation in general.

Our working model involves Ipl1 exclusion from the nucleus through an indirect interaction with FUS, leading to reduced levels of H3S10ph. Our data suggest this mechanism involves Nop1, as its knockdown restores H3S10ph levels and alleviates growth suppression. It is possible that Nop1 interacts—either directly or indirectly—with Ipl1. We postulate that H3K14ac levels are lowered through histone crosstalk with H3S10ph. In a parallel mechanism, Rtt109 mislocalization leads to a decrease in H3K56ac levels. This second mechanism is unaffected by Nop1 knockdown. Moreover, Rrp5 knockdown yeast maintains decreased histone PTM levels in the context of FUS overexpression but displays an alleviation of FUS’s growth suppression. Taken together, this suggests that FUS may lead to growth suppression and histone PTM dysregulation through two or more separate pathways. As another option, histone PTM alterations might be occurring upstream of Rrp5′s involvement in growth suppression. In sum, histone PTM dysregulation and interactions between FUS, Rrp5, and Nop1 result in negative cellular outcomes such as cellular toxicity and PTM dysregulation suggestive of errors in rRNA biogenesis, processing, and heat shock responses in the context of FUS proteinopathy. Furthermore, prior work investigating changes to HMEs in the context of the native yeast prions Rnq1 and Swi1 revealed separate histone PTM landscapes from that noted in the FUS overexpression model used here [99]. This suggests that FUS’ indirect and direct binding partners may be unique to FUS overexpression and not a generalized response to the overall protein aggregation and amyloids. While it is important to note that our studies were limited to yeast models, and thus, mechanistic verification in other model systems is still necessary, our results underscore histone modifiers as potential targets for pharmaceutical intervention in the treatment of ALS/FTD. As these specific pathways are conserved in humans, we propose that these interactions and alterations can be extrapolated to a human model connecting the epigenome and interactome to motor neuron degeneration and death. A schematic representation of the potential mechanisms linking FUS proteinopathy to the epigenome is shown in Figure 6.

## 4. Conclusions

We have illuminated the direct and indirect interactions connecting FUS proteinopathy to the epigenome. We have shown that FUS proteinopathy is linked to the mislocalization of the histone kinase Ipl1 and histone acetyltransferase Rtt109 to the cytoplasm, concurring with a reduction in the H3S10ph and H3K56ac levels. In contrast, the levels and the cellular localization of the histone acetyltransferase Gcn5 are unchanged; therefore, the level of H3K14ac might be lowered through histone crosstalk. We uncovered various proteins belonging to FUS’ yeast interactome, such as Rrp5 and Nop1. The dysregulation of H3S10ph, H3K14ac, and H3K56ac, as well as interactions between FUS, Rrp5, and Nop1 potentially contributed to the FUS pathological mechanism in various distinct ways. Our investigation also raises several interesting questions. For instance, how is Rtt109 excluded from the nucleus? Does crosstalk between H2AQ105me and H3K56ac play a role in Rtt109 mislocalization [96]? It is possible that nucleocytoplasmic transport leads to Rtt109 redistribution [52]? Furthermore, how do FUS binding partners contribute to FUS pathology? Of particular interest is the potential dual mechanism of FUS where the RNA-binding protein Rrp5 is involved in cytotoxicity while Nop1 is involved in both cytotoxicity and PTM dysregulation. Lastly, what is the role of rRNA biogenesis in ALS/FTD? Novel chemical and genetic intervention strategies utilizing ‘epidrugs’ and epigenomic editing aimed at histone modifiers such as Ipl1 (Aurora B kinase), Nop1 (Fibrillarin), and p300/CBP could potentially improve cell survival in the context of neurodegenerative disease [28,100,101,102,103,104,105]. Ultimately, our results offer some details on how protein aggregation links to negative cellular outcomes, highlight the contribution of histone modifiers to FUS ALS/FTD and other neurodegenerative diseases, and reveal novel targets for therapeutic intervention.

## Data Availability

The original contributions presented in this study are included in the article/Supplementary Materials. Further inquiries can be directed to the corresponding author(s). The following files are available free of charge: tables showing the primers used, putative binding partners, and GO and KEGG annotations (Supplementary Tables S1–S3) (PDF), and figures for the DNA gels for insert creation and insertion verification, silver staining, GO annotations, the Western blot analysis of Co-IPs, and glucose serial dilution assays (Supplementary Figures S1–S5) (PDF).

## Supplementary Materials

The following supporting information can be downloaded at: https://www.mdpi.com/article/10.3390/jof11010058/s1, Table S1: Primers used for PCR Targeting; Table S2: Putative FUS Binding Partners in Yeast. Gene Symbol; Table S3: Gene Ontology and KEGG Pathway terms associated with putative; Figure S1: Verification of FLAG tag insertion; Figure S2: Unique proteins co-immunoprecipitate with FUS; Figure S3: Top GO Biological Process and Molecular Function Annotations Among FUS Putative Binding Partners; Figure S4: DAmP does not affect yeast growth on Glucose Media in the Context of FUS Overexpression; Figure S5: DAmP does not affect yeast growth on Glucose Media in the Context of TDP-43 Overexpression.

## Abbreviations

ALS	Amyotrophic Lateral Sclerosis
Co-IP	co-immunoprecipitation
FTD	Frontotemporal Dementia
HAT	histone acetyltransferase
HDAC	histone deacetylase
HMEs	Histone Modifying Enzymes
ICC	immunocytochemistry
MS	mass spectrometry
PCR	polymerase chain reaction
PTM	post-translational modification
