# Supplementary material for: Direct and Indirect Protein Interactions Link FUS Aggregation to Histone Post-Translational Modification Dysregulation and Growth Suppression in an ALS/FTD Yeast Model
Source: J Fungi (Basel). 2025 Jan 14;11(1):58. doi: 10.3390/jof11010058 (PMC11766905; doi:10.3390/jof11010058)
Supplement: Supplementary file 1 [file jof-11-00058-s001.zip › jof-3372848-supplementary.pdf]

Supplementary Table 1. Primers used for PCR Targeting.

**Primers Used for Insert Creation**		
Gcn5-FLAG	Forward	TAAAGTAAAAGAAATACCTGAATATTCTCACCTTATTGATGGTGGAGGCTCTAGAGACTA
	Reverse	TCGAAAGGAATAGTAGCGGAAAAGCTTCTTCTACGCATTATTAAACTGGATGGCGGCGTT
Rtt109-FLAG	Forward	GCTAAAACCGCGTAAAAAAGCTAAAGCCTTGCCTAAAACTGGTGGAGGCTCTAGAGACTA
	Reverse	TCGATGCTACATACGTGTACTAAATAATAAATATCAATATGTATCATTAAACTGGATGGCGGCGTT
Ipl1-FLAG	Forward	GATACTAAGAAACAAGCCCTTTTGGGAAAATAAGCGGTTAGGTGGAGGCTCTAGAGACTA
	Reverse	TCGATGCTACATACGTGTACTAAATAATAAATATCAATATGTATCATTAAACTGGATGGCGGCGTT
**Primers Used for Insert Verification**		
Universal Reverse	TTAAACTGGATGGCGGCGTT	
Gcn5 Forward	GCCGAATGTACAATGGCGAG	
Rtt109 Forward	AAATCAGCCGGTTCCTGCAA	
Ipl1 Forward	TAGAATGCGCCTTGGAGACG	

*All primers are shown in 5’ to 3’ direction.

Supplementary Table 2. Putative FUS Binding Partners in Yeast.

Gene Symbol	Protein Description	UniProt Accession Number	Molecular weight (kDa)
**ACC1**	Acetyl-CoA Carboxylase	Q00955	250.2
**BEM2**	Rho GTPase Activating Protein	P39960	245.3
**DBP1**	ATP-dependent RNA helicase	P24784	112.9
**DBP2**	ATP-dependent RNA helicase	P24783	61
**DED1**	ATP-dependent RNA helicase	P06634	65.5
**FAS2**	Fatty acid synthase	P19097	206.8
**HHF1**	Histone H4	P02309	11.4
**HSC82**	Cytoplasmic chaperone – Hsp90	P15108	80.8
**MCM4**	Helicase component of MCM	P30665	104.9
**MSS116**	Transcription elongation factor	P15424	76.2
**MYO2**	Type V myosin motor	P19524	180.6
**NOP1**	Histone methyltranserfase	P15646	34.4
**NOP56**	C/D snoRNP complex component	Q12460	56.8
**OLA1**	P-loop APTase	P38219	44.1
**PAB1**	Poly(A) binding protein	P04147	64.3
**PET9**	Mitochondrial ADP/ATP carrier	P18239	34.4
**PMA1**	P2-type H+ ATPase	P05030	99.6
**PMA2**	P2-type H+ ATPase	P19657	102.1
**PRP43**	RNA helicase	P53131	87.5
**RPO21**	RNA polymerase II subunit	P04050	191.5
**RRP5**	RNA binding protein	Q05022	193
**SAM1**	S-adenosylmethionine synthase	P10659	41.8

**SAM2**	S-adenosylmethionine synthase	P19358	42.2
**SSA1**	Nuclear transport ATPase – Hsp70	P10591	69.6
**SSA2**	Hsp70 ATP-binding protein	P10592	69.4
**SSB2**	Ribosome chaperone ATPase	P40150	66.6
**TCB1**	Lipid-binding ER protein	Q12466	133.5
**TCB3**	Cortical ER protein	Q03640	171
**TEF1**	Translational elongation factor	P02994	50
**TOR1**	Kinase subunit of TORC1	P35169	281
**TY1A-OL**	Structural constituent of VLPs	Q92392	49
**TY1B-PR1**	Polyprotein processed to make Gag, RT, PR and IN	P0C2I9	198.4
**URA2**	Carbamoylphosphate synthetase	P07259	244.9
**UTP10**	Component of small subunit processome	P42945	200
**UTP22**	Component of small subunit processome	P53254	140.4
**VPS1**	GTPase required or vacuolar sorting	P21576	78.7
**YHB1**	Nitric oxide oxioreductase	P39676	44.6
**YHR020W**	Prolyl-tRNA synthetase	P38708	77.3
**YLR419W**	Putative helicase	Q06698	162.9

Supplementary Table 3. Gene Ontology and KEGG Pathway terms associated with putative FUS binding proteins.

GO Biological Pathway	
Term	P-Value
GO:0006364: rRNA processing	3.50E-04
GO:0031167: rRNA methylation	5.80E-04
GO:0010501: secondary structure unwinding	7.10E-04
GO:0042254: ribosome biogenesis	1.80E-03
GO:0006556: S-adenosylmethionine biosynthetic process	2.00E-02
GO:0090344: negative regulation of cell aging	2.00E-02
GO:0000451: rRNA 2'-O-methylation	2.60E-02
GO:0042759: long-chain fatty acid biosynthetic pathway	2.60E-02
GO:0006885: regulation of pH	3.30E-02
GO:0090158: endoplasmic reticulum membrane organization	3.90E-02
GO:0000390: spliceosomal complex disassembly	3.90E-02
GO:0006413: translational initiation	4.30E-02
GO:0006555: methionine metabolic process	4.60E-02
GO:0060304: regulation of phosphatidylinositol dephosphorylation	4.60E-02
GO:0051453: regulation of intracellular pH	5.20E-02
GO:0010468: regulation of gene expression	7.70E-02
GO:0000462: maturation of SSU-rRNA from tricistronic rRNA transcript	8.10E-02
GO:0006409: tRNA export from the nucleus	8.90E-02
GO:0030490: maturation of SSU-rRNA	9.50E-02

GO Molecular Function	
Term	P-Value
GO:0005524: ATP binding	1.60E-09
GO:0000166: nucleotide binding	1.90E-09
GO:0004004: ATP-dependent RNA helicase activity	1.00E-05
GO:0003729: mRNA binding	7.00E-05
GO:0004386: helicase activity	1.00E-04
GO:0016887: ATPase activity	3.80E-04
GO:0003676: nucleic acid binding	1.30E-03
GO:0003723: RNA binding	9.30E-03
GO:0046872: metal ion binding	1.00E-02
GO:0030515: snoRNA binding	1.10E-02
GO:0005544: calcium-dependent phospholipid binding	1.50E-02
GO:0004478: methionine adenosyltransferase	1.50E-02
GO:0033592: RNA strand annealing activity	2.30E-02
GO:0051082: unfolded protein binding	2.40E-02
GO:0016787: hydrolase activity	2.70E-02
GO:0008553: hydrogen-exporting ATPase activity, phosphorylative mechanism	3.80E-02
GO Cellular Component	
Term	P-Value
GO:0010494: cytoplasmic stress granule	2.20E-05
GO:0005739: mitochondrion	4.00E-05
GO:0030686: 90S preribosome	1.50E-04
GO:0032040: small-subunit proccessome	3.30E-04
GO:0005886: plasma membrane	3.00E-03
GO:0005654: nucleoplasm	3.00E-03

GO:0000329: fungal-type vacuole membrane	1.10E-02
GO:0005844: polysome	1.80E-02
GO:0005737: cytoplasm	1.90E-02
GO:0036464: cytoplasmic ribonucleoprotein granule	2.60E-02
GO:0031428: box C/D snoRNP complex	3.80E-02
GO:0005832: chaperonin-containing T-complex	6.90E-02
GO:0005730: nucleolus	9.70E-02
KEGG Pathway	
Term	P-Value
sce03400: Spliceosome	7.10E-03
sce04141: Protein processing in endoplasmic reticulum	5.30E-02
sce03008: Ribosome biogenesis in eukaryotes	5.80E-02

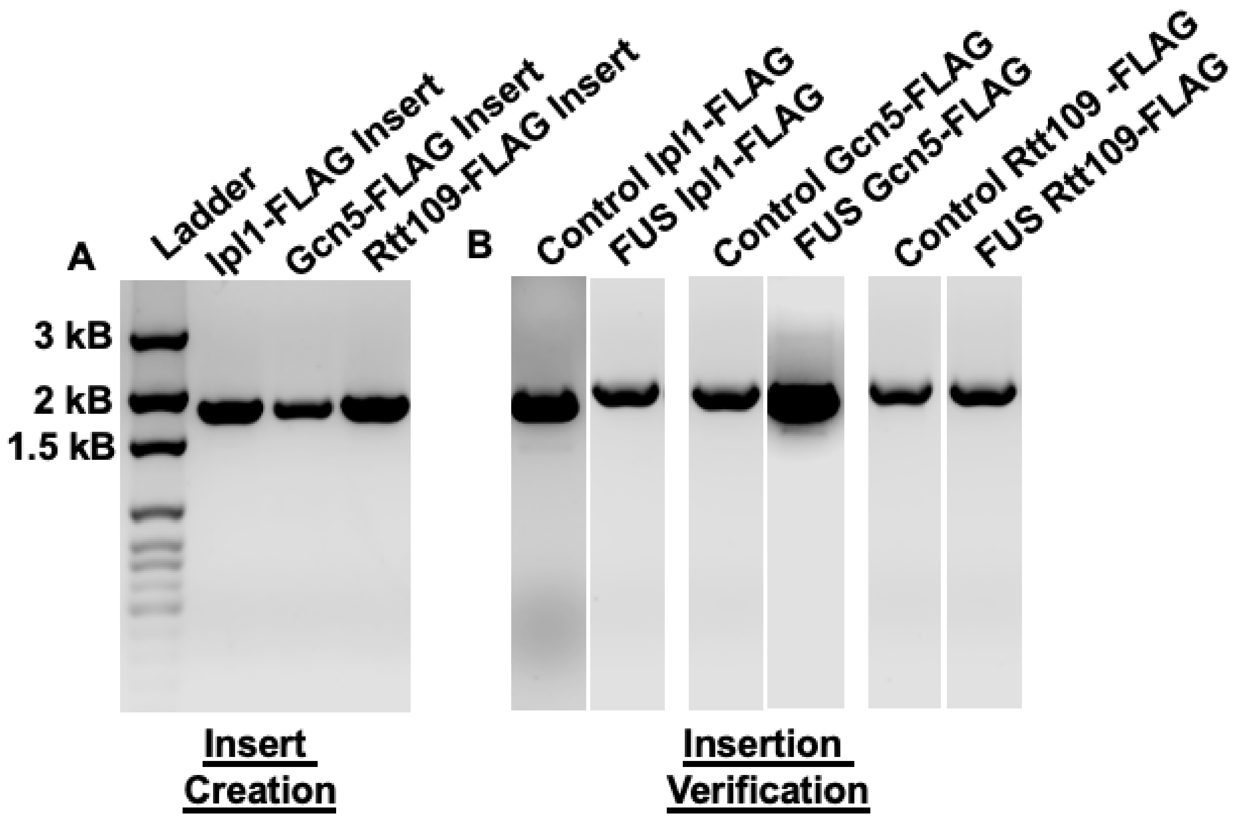

Supplementary Figure 1. Verification of FLAG tag insertion. A) Creation of constructs was verified by 1% agarose gel electrophoresis followed by staining with ethidium bromide. B) Insertion of FLAG at the Ipl1, Gcn5 and Rtt109 loci in FUS and control yeast was verified by PCR amplification visualized by 1% agarose gel electrophoresis followed by staining with ethidium bromide. n = 3.

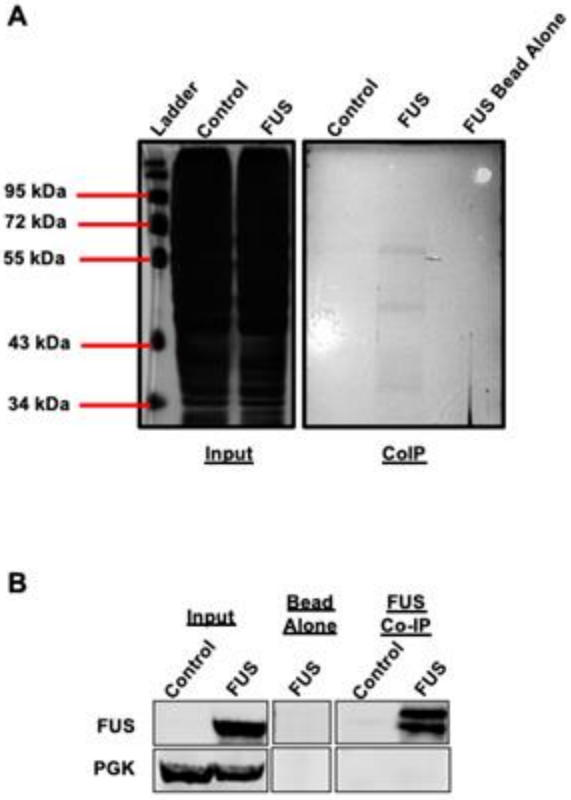

Supplementary Figure 2. Unique proteins co-immunoprecipitate with FUS. A FUS antibody was used as bait in a Co-IP experiment. The recovered proteins were separated by gel electrophoresis and Silver stained for visualization A) Several discrete bands are present in the FUS Co-IP compared to the vector and bead alone controls, including a large band near the 34 kDa region. B) FUS co-immunoprecipitation verification via Western blotting. n = 3.

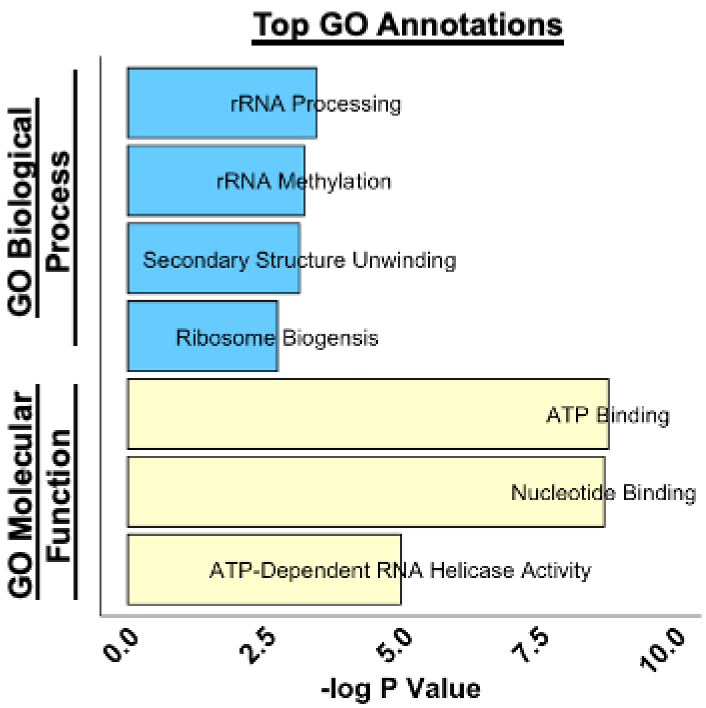

Supplementary Figure 3. Top GO Biological Process and Molecular Function Annotations Among FUS Putative Binding Partners.

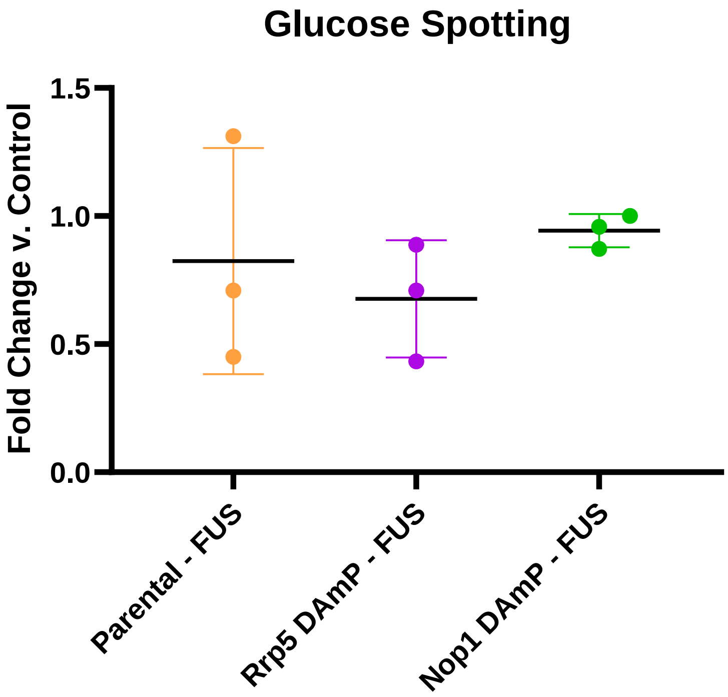

Supplementary Figure 4. DAmP does not affect yeast growth on Glucose Media in the Context of FUS Overexpression. Column scatterplot represents the densitometric measurement of cell density (middle spot) compared to control yeast (middle spot of control yeast on glucose plates in Figure 4a from Main Text). n = 3 for each experiment.

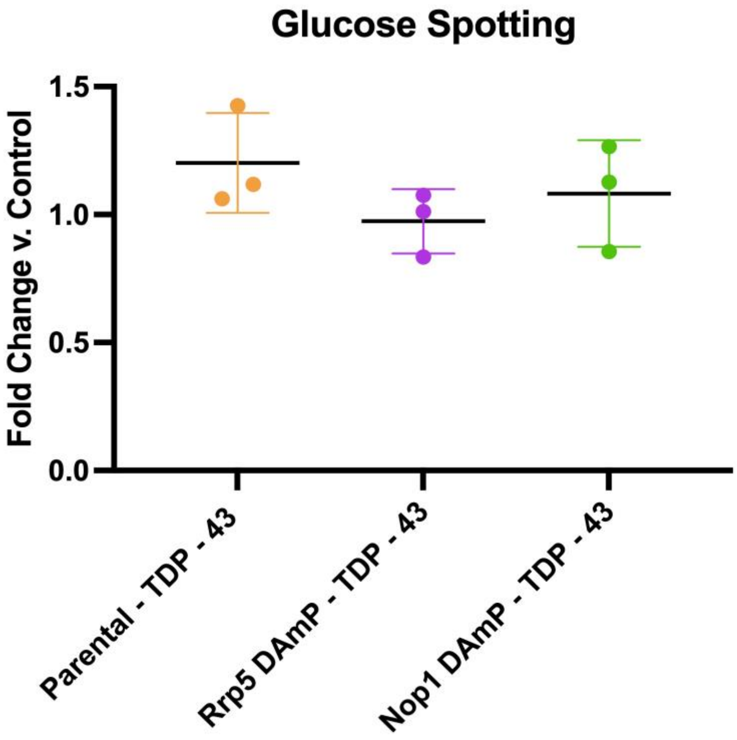

Supplementary Figure 5. DAmP does not affect yeast growth on Glucose Media in the Context of TDP-43 Overexpression Column scatterplot represents the densitometric measurement of cell density (middle spot) compared to control yeast (middle spot of control yeast on glucose plates in Figure 5a from Main Text). n = 3 for each experiment.
